# A case of multiple metastatic sarcomatoid renal cell carcinoma with complete response to nivolumab

**DOI:** 10.1002/cnr2.1356

**Published:** 2021-03-03

**Authors:** Masayuki Tomioka, Keita Nakane, Kaori Ozawa, Koji Iinuma, Natsuko Suzui, Tatsuhiko Miyazaki, Takuya Koie

**Affiliations:** ^1^ Department of Urology Gifu University Graduate School of Medicine Gifu Japan; ^2^ Department of Urology, Japanese Red Cross Takayama Hospital Takayama Japan

**Keywords:** complete response, multiple metastases, nivolumab, renal cell carcinoma, sarcomatoid carcinoma

## Abstract

**Background:**

Sarcomatoid renal cell carcinoma (SRCC) is associated with poor prognosis. Although there is no standard treatment for SRCC, recent studies have reported the effectiveness of immune checkpoint inhibitors.

**Case:**

An 82‐year‐old Japanese man presented to our hospital with an incidental right renal tumor. Abdominal computed tomography (CT) showed an exophytic tumor in the right kidney with suspected right iliopsoas muscle invasion. Laparoscopic right radical nephrectomy was performed. Histopathological diagnosis revealed a clear cell RCC with a spindle cell carcinoma component. CT performed 3 months after surgery revealed multiple bilateral lung metastases and local recurrence. Although the patient received tyrosine‐kinase inhibitors for treating multiple metastases, the lung metastases continued to gradually increase, and peritonitis carcinomatosis was observed. Thus, the patient was intravenously administered nivolumab once every 2 weeks. After nivolumab administration, lung metastases, local recurrence, and peritonitis carcinomatosis gradually reduced. After 20 months of nivolumab treatment, the patient achieved a complete response of multiple metastases on CT.

**Conclusion:**

Nivolumab may be used as a treatment option for sarcomatoid renal cell carcinoma with multiple metastases.

## 1 INTRODUCTION

Sarcomatoid renal cell carcinoma (SRCC) accounts for 1% to 5% of all renal neoplasms and is also known as spindle cell carcinoma, anaplastic carcinoma, and carcinosarcoma.^1^, ^2^ Renal cell carcinoma (RCC) with a sarcomatoid component is associated with poor prognosis even for patients diagnosed with stage I or II RCC. Most patients with RCC with a sarcomatoid variant showed local recurrence or distant metastases at diagnosis.^1^, ^2^ In general, the oncological outcomes of SRCC are unfavorable, and the median survival of SRCC patients has been reported to be between 5 and 12 months.^3^ In addition, the time from nephrectomy to cancer recurrence was significantly shorter in patients with SRCC than in those with non‐SRCC (18.8 vs 42.9 months; *P* < .0001).^4^ Recently, some reports have suggested that immune checkpoint inhibitors (ICIs) are particularly effective for SRCC.^5^, ^6^, ^7^, ^8^ In this study, we reported a stage IV SRCC patient, who achieved complete response to nivolumab treatment.

## 2 CASE

**FIGURE 1:**
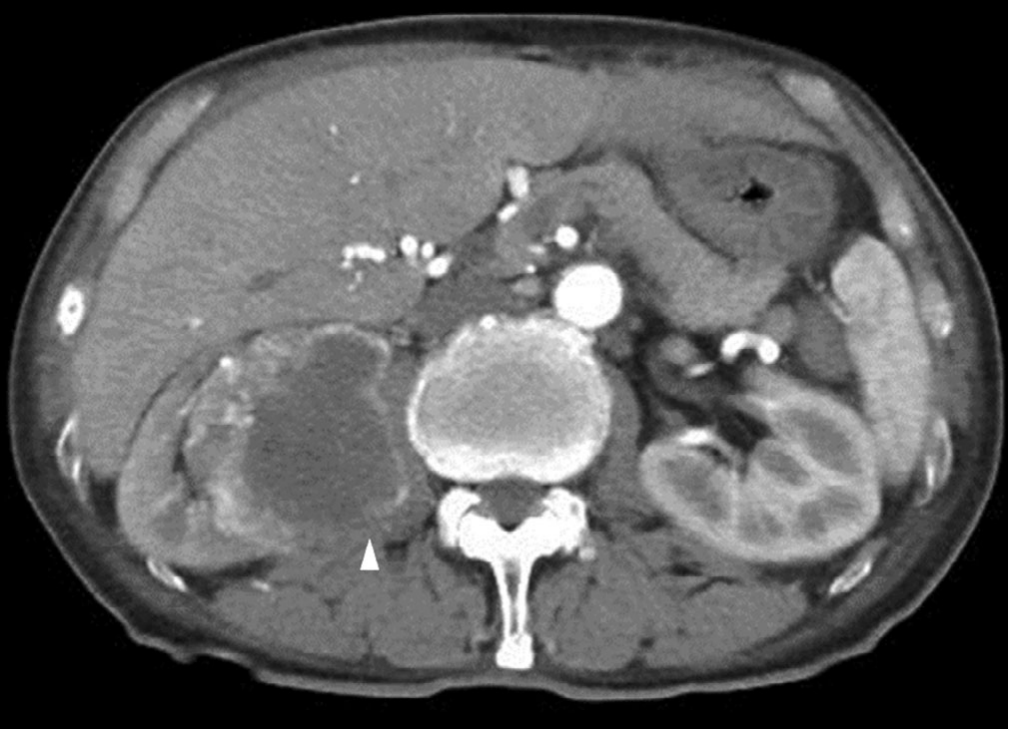
Abdominal computed tomography showing a right renal tumor in the lower pole with suspected invasion to the right iliopsoas muscle (arrow)

An 82‐year‐old Japanese male patient presented with an incidental right renal tumor at our hospital. Abdominal computed tomography (CT) showed an exophytic tumor measuring 6.2 cm × 6.3 cm in the lower pole of the right kidney with suspected right iliopsoas muscle invasion and right adrenal gland metastasis (Figure 1). The patient did not show lymph node involvement or distant metastases at this point.

**FIGURE 2:**
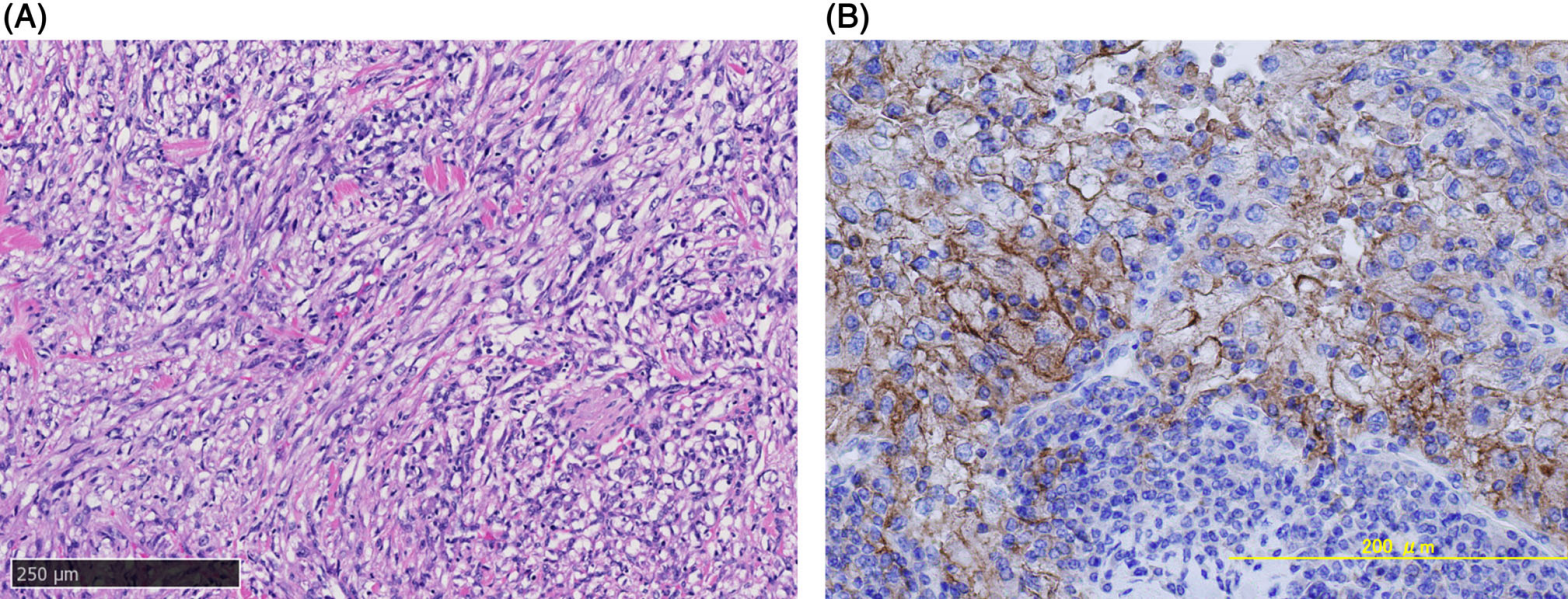
(A) Histopathological findings showing clear cell renal cell carcinoma with a spindle cell carcinoma component. (B) Immunohistochemistry analysis using anti‐programmed death‐ligand 1 (PD‐L1) antibody (clone 28‐8) showed that PD‐L1 was expressed in 40% of the surgical specimens

Laparoscopic right radical nephrectomy with right adrenalectomy was performed. Histopathological diagnosis revealed clear cell RCC with a spindle cell carcinoma component (40%) (Figure 2A). Immunohistochemistry analysis using anti‐programmed death‐ligand 1 (PD‐L1) antibody (clone 28‐8) showed that PD‐L1 was expressed 25% in tumor positive score (TPS), 20% in immune cells (IC), and 40 in combined positive score (CPS) (Figure 2B).

**FIGURE 3:**
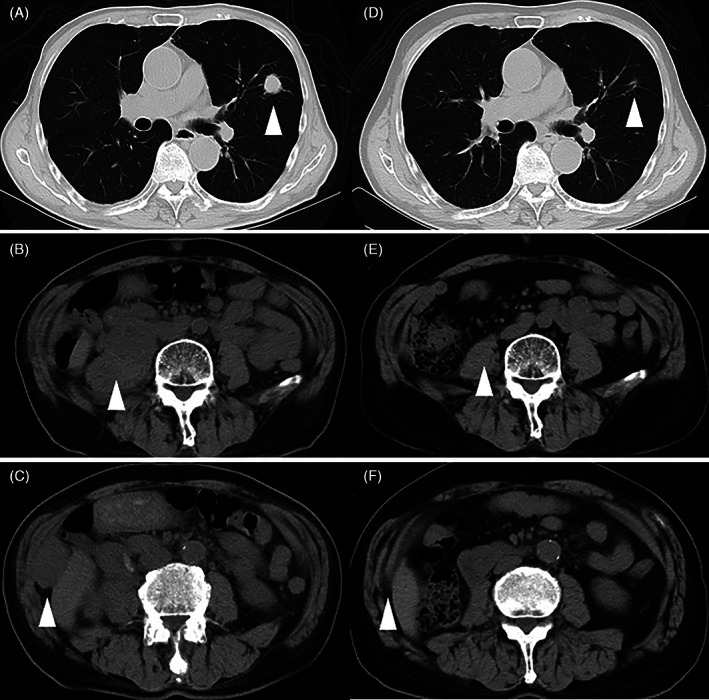
Computed tomography (CT) taken before (A–C) and after the administration of nivolumab (D–F) (arrows). (A–C) CT shows lung metastasis (A), local recurrence (B), and peritonitis carcinomatosis (C). (D–F) After 24 months of nivolumab treatment, the patient achieved a complete response with multiple metastases on CT (arrows)

CT scans 3 months after the surgery revealed multiple bilateral lung metastases and local recurrence. Although we did not perform needle biopsies for metastatic lesions, we considered that multiple metastases most likely developed from the SRCC owing to the patient's clinical course after surgery. The patient was classified as having intermediate risk according to the International Metastatic RCC Database Consortium risk classification and the Memorial Sloan‐Kettering Cancer Center prognostic model because he fulfilled the criteria of the hemoglobin level being below the lower limit of the normal range and less than 1 year from time of diagnosis to systemic therapy.^6^, ^7^ The patient had advanced age and was worried that sunitinib‐related adverse events would lower performance status. Therefore, sunitinib was administered at a dose of 25 mg orally once daily for 2 weeks on and 1 week off in each 3‐week cycle. It was well‐tolerated by the patient, who showed only grade 2 adverse events. At that time, five lung metastases were identified as target lesions. However, the diameter of targeted lung metastases increased from 5.6 to 7.4 cm (32% increase) in 2 months after sunitinib administration. Therefore, treatment was switched to axitinib, which was administered 5 mg twice daily. However, lung metastases continued to gradually increase, and peritonitis carcinomatosis was observed 2 months after the initiation of axitinib. Thus, the patient was then administered nivolumab (240 mg) intravenously once every 2 weeks. After nivolumab administration, lung metastases, local recurrence, and peritonitis carcinomatosis reduced gradually. After 20 months of nivolumab treatment (24 months after the diagnosis of RCC), the patient achieved complete response with multiple metastases on CT, and treatment‐related adverse events were not seen during nivolumab administration (Figure 3).

## 3 DISCUSSION

In recent years, the treatment for metastatic RCC has dramatically changed. The prognosis of RCC patients with lymph node involvement or distant metastases receiving ICIs has also changed. Several patients have shown a decreased number of metastatic sites, and some patients have achieved complete response to ICI treatment. According to a phase III trial comparing nivolumab with everolimus in patients with previously treated advanced RCC (CheckMate 025), median progression‐free survival (PFS) was 4.6 months with nivolumab and 4.4 months with everolimus (*P* = .11), even though overall survival (OS) in patients who received nivolumab was significantly longer than those who were administered everolimus (*P* = .0018).^9^ In an open‐label phase III trial (KEYNOTE‐426), patients with advanced RCC who received pembrolizumab plus axitinib had significantly longer OS and PFS and a higher objective response rate than those who received sunitinib only.^8^ In the phase III JAVELIN Renal 101 trial, PFS was significantly longer with avelumab plus axitinib than with sunitinib among patients who received these agents as first‐line treatments for advanced RCC.^10^ Based on the results of IMmotion151 trial for untreated metastatic RCC, the median PFS was 11·2 months in the atezolizumab plus bevacizumab group vs 7·7 months in the sunitinib group, in the PD‐L1 positive population (*P* = .022).^11^ In CheckMate 9ER, nivolumab plus cabozantinib demonstrated superiority over sunitinib by doubling the PFS time, doubling the OS rate, and significantly improving OS for advanced RCC.^12^ From these results, combination therapy may have several advantages with oncological outcomes in advanced or metastatic RCC than nivolumab monotherapy.

Conversely, SRCC has a more aggressive disease biology and poorer oncological outcomes than other RCCs.^5^ In addition, the optimal treatment regimens for SRCC remain unknown according to several guidelines. According to molecular‐targeting therapy for metastatic RCC with sarcomatoid differentiation, Golshayan et al reported that treatment with sunitinib, sorafenib, or bevacizumab, achieved partial response of metastatic disease in 19% of the patients who had mainly clear cell carcinoma with >20% sarcomatoid component in the primary tumors.^13^ In addition, median tumor shrinkage, PFS, and OS were 2%, 5.3, and 11.8 months, respectively.^13^ In a phase II trial for SRCC patients who received the combination of capecitabine, gemcitabine, and bevacizumab, the median OS and PFS were 12 and 5.5 months, respectively.^14^ Recent randomized controlled trials using ICIs for RCC treatment have reported that RCC with a sarcomatoid component clearly showed improved oncological outcomes, including OS or PFS rates.^3^, ^5^, ^6^, ^8^ The *post‐hoc* analysis of CheckMate‐214 study evaluated the utility of nivolumab plus ipilimumab (NIVO+IPI) vs sunitinib in patients with SRCC.^3^ The median OS in patients who received NIVO+IPI was significantly longer than that in those who received sunitinib (*P* = .0004); PFS benefits with NIVO+IPI were similarly observed (*P* = .0093).^3^ While only 23.1% of the SRCC patients who received sunitinib achieved objective response, 60.8% of those who received NIVO+IPI achieved objective response (*P* < .0001).^3^ Additionally, the patients who received NIVO+IPI, unlike those who received sunitinib, achieved complete response (18.9% vs 3.1%, respectively). On the other hand, a retrospective study from Roswell Park Comprehensive Cancer Center reported the efficacy of ICIs in patients with metastatic SRCC.^15^ Although the median OS in patients who received ICs (CPI arm) was 33.8 months compared to 8.8 months in those who received non‐immunotherapy (no CPI arm), 30% of the patients in CPI arm and 25% of those in non CPI arm were alive after a median follow‐up of 35 months.^14^ Therefore, these results suggest that the combination therapy, including ICIs, may improve oncological outcomes in patients with advanced or metastatic RCC.

The reason why patients who received ICIs showed improved oncological outcomes remains unclear. It is possible that the patient had PD‐L1 expression of 25% in TPS, 20% in IC, and 40 in CPS. Recently, it has been found that there is higher PD‐L1 expression and higher PD1‐ and CD8‐positive cell density in SRCC than in grade 4 clear cell RCC.^15^ In the CheckMate 214 population, PD‐L1 was a prognostic factor for poor oncological outcomes and was associated with more advanced disease.^6^, ^16^ These data may support the use of ICIs in patients with SRCC regardless of the tumor PD‐L1 expression level.^3^ Further large‐scale clinical or case‐control observational studies are needed to validate the utility of ICIs as a treatment option for SRCC. In addition, molecular analyses may further characterize the immune response in SRCCs.

## 4 CONCLUSION

We reported an SRCC patient who showed multiple metastases. This was a case of multiple metastatic progressive SRCC, showing a complete response to nivolumab treatment. ICIs may be used a treatment option for SRCC with lymph node involvement or distant metastases.

## CONFLICT OF INTEREST

The authors declare no conflicts of interest.

## AUTHOR CONTRIBUTIONS

All authors had full access to the data in the study and take responsibility for the integrity of the data and the accuracy of the data analysis. *Conceptualization*, M.T., T.K.; *Investigation*, K.N., K.O., K.I.; *Formal Analysis*, N.S.,T.M.; *Writing‐Original Draft*, M.T.; *Writing–Review & Editing*: T.K.

## ETHICS STATEMENT

Informed consent was obtained to publish this report.

## Data Availability

The data that support the findings of this study are available from the corresponding author upon reasonable request.
